# In-depth hepatoprotective mechanistic study of *Phyllanthus niruri*: *In vitro* and *in vivo* studies and its chemical characterization

**DOI:** 10.1371/journal.pone.0226185

**Published:** 2020-01-15

**Authors:** Marwa I. Ezzat, Mona M. Okba, Sherif H. Ahmed, Hossny A. El-Banna, Abdelbary Prince, Shanaz O. Mohamed, Shahira M. Ezzat

**Affiliations:** 1 Pharmacognosy Department, Faculty of Pharmacy, Cairo University, Kasr El-Ainy Street, Cairo, Egypt; 2 Department of Biochemistry, Faculty of Agriculture, Cairo University, Giza, Egypt; 3 Department of Pharmacology, Faculty of Veterinary Medicine, Cairo University, Giza, Egypt; 4 Department of Biochemistry, Faculty of Veterinary Medicine, Cairo University, Giza, Egypt; 5 School of Pharmaceutical Sciences, Universiti Sains Malaysia, Penang, Malaysia; 6 Department of Pharmacognosy, Faculty of Pharmacy, October University for Modern Sciences and Arts (MSA), Giza, Egypt; Jadavpur University, INDIA

## Abstract

*Phyllanthus niruri* L. is a widespread tropical plant which is used in Ayurvedic system for liver and kidney ailments. The present study aims at specifying the most active hepatoprotective extract of *P*. *niruri* and applying a bio-guided protocol to identify the active compounds responsible for this effect. *P*. *niruri* aerial parts were extracted separately with water, 50%, 70% and 80% ethanol. The cytoprotective activity of the extracts was evaluated against CCl_4_-induced hepatotoxicity in clone-9 and Hepg2 cells. Bioassay-guided fractionation of the aqueous extract (AE) was accomplished for the isolation of the active compounds. Antioxidant activity was assessed using DPPH (1, 1-diphenyl-2-picrylhydrazyl) radical scavenging method and ferric reducing antioxidant power (FRAP). The *in vivo* hepatoprotective activity of AE was evaluated in CCl_4_-induced hepatotoxicity in rats at different doses after determination of its LD_50_. Pretreatment of clone-9 and Hepg2 with different concentrations of AE (1, 0.1, 0.01 mg/ml) had significantly reduced the levels of alanine aminotransferase (ALT) and aspartate aminotransferase (AST) against CCl_4_ injures, and restored the activity of the natural antioxidants; glutathione (GSH) and superoxide dismutase (SOD) towards normalization. Fractionation of AE gave four fractions (I-IV). Fractions I, II, and IV showed a significant *in vitro* hepatoprotective activity. Purification of I, II and IV yielded seven compounds; corilagin **C1**, isocorilagin **C2**, brevifolin **C3**, quercetin **C4**, kaempferol rhamnoside **C5,** gallic acid **C6**, and brevifolin carboxylic acid **C7**. Compounds **C1**, **C2**, **C5**, and **C7** showed the highest (*p*< 0.001) hepatoprotective potency, while **C3**, **C4**, and **C6** exhibited a moderate (*p*< 0.001) activity. The AE exhibited strong antioxidant DPPH (IC_50_ 11.6 ± 2 μg/ml) and FRAP (79.352 ± 2.88 mM Ferrous equivalents) activity. *In vivo* administration of AE in rats (25, 50, 100 and 200 mg/kg) caused normalization of AST, ALT, alkaline phosphatase (ALP), lactate dehydrogenase (LDH), total cholesterol (TC), triglycyrides (TG), total bilirubin (TB), glucose, total proteins (TP), urea and creatinine levels which were elevated by CCl_4_. AE also decreased TNF-α, NF-KB, IL-6, IL-8, IL10 and COX-2 expression, and significantly antagonizes the effect of CCl_4_ on the antioxidant enzymes SOD, catalase (CAT), glutathione reductase (GR), and glutathione peroxidase (GSP). The histopathological study also supported the hepatoprotective effect of AE. *P*. *niruri* isolates exhibited a potent hepatoprotective activity against CCl_4_-induced hepatotoxicity in clone-9 and Hepg2 cell lines through reduction of lipid peroxidation and maintaining glutathione in its reduced form. This is attributable to their phenolic nature and hence antioxidative potential.

## Introduction

Liver injury, caused by viruses, drugs and chemicals, is a significant toxicological problem [1–3]. The damage is associated with metabolic and synthetic dysfunctions which can lead to fatal complications [4]. CCl_4_-induced acute liver injury is the best characterized system of xenobiotic-induced hepatotoxicity and a common screening model for evaluation of the hepatoprotective potential of drugs [5]. The pathogenesis of the damage is multivariate [6] involving propagation of a chain of free radicals, leading to lipid peroxidation and destruction of cellular membranes, followed by triggering the inflammatory response of the body [7, 8]. In spite of the fact that advances in understanding of the liver damage molecular mechanisms are achieved, there are still limited effective hepatoprotective interventions. Thus, herbal alternatives drew much attention as a safe solution for this problem.

The *Phyllanthus* genus contains over 600 species distributed throughout the tropical and subtropical regions of the world. The plants of genus *Phyllantus* have long been used to treat liver diseases [9]. A wide number of experimental studies have demonstrated the hepatoprotective potential of *Phyllantus* plants in *in vitro* and *in vivo* systems [10–12].

*P*. *niruri* has a good reputation in herbal medicine systems such as Indian Ayurveda, Traditional Chinese Medicine and Indonesian Jamu for over 2000 years. *P*. *niruri* has been used as a remedy for many ailments such as dyspepsia, influenza, diuretics, vaginitis, hyperglycaemia, jaundice and removing kidney stones [13]. *P*. *niruri* is named in Spanish as Chanca Piedra, this means stone breaker, as it was used as an excellent remedy for gallstones and kidney stones elimination [14]. It is named Quebra Pedra in Brazilian herbal medicine, where it is considered an effective remedy for urinary and bladder disorders as well as hepatic disorders and hyperglycemia. *P*. *niruri* is used as a remedy for asthma, bronchitis, coughs in India, for this reason it is named Pitirishi or Budhatri [13]. *P*. *niruri* was specifically tested for its hepatoprotective [15–17], antioxidant [18–20], antihyperuricemic [21] and lipid lowering activities [22]. Its actions were evaluated on various organs including liver, kidneys and testes [23].

This study aims at optimizing a method for extraction of *P*. *niruri* based on evaluation of the *in vitro* hepatoprotective activity on rat liver normal cell line (clone-9) and human liver hepatoma cells (Hepg2). In addition, this study focuses on applying a bioassay guided fractionation of the active extract to identify the active fraction and then the active isolate, and finally to confirm the hepatoprotective activity of the active extract via evaluation of its *in vivo* hepatoprotective activity against CCl_4_-induced hepatotoxicity in rats.

## Materials and methods

### General

Silica gel 60 (70–230 mesh ASTM; Fluka, Steinheim, Germany), Sephadex LH 20 (Pharmacia, Stockholm, Sweden) and Diaion HP-20 AG (75–150 μm, Mitsubishi Chemical Industries Co. Ltd) were used for column chromatography. Thin-layer chromatography (TLC) was performed on silica gel GF_254_ precoated plates (Fluka, Steinheim, Germany). The used solvent systems were **S1** [methylene chloride-methanol-formic acid (9.5:0.5:0.2 v/v/v)], **S2** [methylene chloride-methanol-formic acid (8.5:1.5:0.2 v/v/v)] and **S3** [methylene chloride-methanol-formic acid (7:3:0.2 v/v/v)]. Bruker NMR was used for ^1^H-NMR (400 MHz) and ^13^C-NMR (100 MHz) measurements. The NMR spectra were recorded in deuterated dimethyl sulfoxide (DMSO-*d6*) and chemical shifts were given in *δ* (ppm) relative to TMS as internal standard. CCl_4_, Ellman's reagent and thiobarbituric acid (TBA) were purchased from Sigma Chemical Co., St Louis, MO, USA. *n*-Butanol, dipotassium hydrogen phosphate, potassium dihydrogen phosphate and trichloroacetic acid were purchased from El-Gomhoreya Chemical Co, Cairo, Egypt. All other chemicals were of highest grade commercially available materials.

### Plant material

The aerial parts of *Phyllanthus niruri* L. were obtained from HCA products Sdn Bhd (922996-T), Blok N1,UPM MTDC Technology Center, Universiti Putra Malaysia 43400 Serdang Selangor, spring 2016. The aerial parts were harvested in the morning and put inside plastic containers during the collecting period then placed in oven at a control temperature of 40–50°C. The drying process took 2–3 hours to ensure that the raw material was dry and no substance of humidity. The dried aerial parts were powdered and placed inside a glass container. The plant was kindly identified in the Forest Research institute, Malaysia. Plant samples were also authenticated by Prof. Dr. Wafaa Amer, Department of Botany, Faculty of Science, Cairo University, Giza, Egypt.

### Preparation of the aqueous extract

The aqueous extract (AE, DA001NW) was obtained from HCA products Sdn Bhd, where the dried powdered aerial parts (40 kg) of *P*. *niruri* were boiled with 200 liters of RO water (water purified with reverse osmosis) for 2h and 30 min. The extract was concentrated in a rotary evaporator for 3 hours at 60°C to 20 liters. The extract was then dried in a spray dryer by heating for 6h and 30 min at a temperature of 120°C and yielded 8 kg powdered extract. The (AE) was tested for potentially toxic or harmful compounds (toxic metals, adulterants, adventitious toxin, foreign materials and residual pesticides) by HCA products Sdn Bhd and that no such compounds were identified in the analysis.

### Preparation of the crude ethanol extracts

The powdered dried *P*. *niruri* aerial parts (50 g powder for each extract) were separately extracted using 50%, 70% and 80% ethanol. The liquid-material ratio was 90:1 in a three-stage procedure (each in a ratio of 30:1), using ultrasonic bath at 60°C for 30 minutes each time. The extracts were concentrated separately using rotary evaporator under reduced pressure (at 40°C) to yield solid residues weighing 5.35, 4.14 and 2.42 g of 50%, 70% and 80% ethanol extracts, respectively.

### *In vitro* hepatoprotective activity

#### Cell culture

Rat liver normal cell line (clone-9) and human liver hepatoma cell line (Hepg2) (VACSERA, Giza, Egypt) were maintained in the tissue culture facility, Faculty of Pharmacy, Ain Shams University, Cairo, Egypt. Cells were grown in Dulbecco’s modified Eagle’s medium (DMEM).

Cells were maintained at 37°C in a 5% CO_2_ atmosphere with 95% humidity. The stock culture was grown in 25 cm^2^ culture flasks and all experiments were carried out in 96 microtitreplates. Cell culture reagents were obtained from Lonza (Basel, Switzerland) [24].

#### CCl_4_ induced toxicity in Clone-9 and Hepg2 cell lines

The protective activities of the samples were examined *in vitro* at three concentrations (1, 0.1, 0.01 mg/mL). Cultured clone-9 and Hepg2 monolayer cells were incubated with the different concentrations of samples. At 1 h after incubation, CCl_4_ was added at a final concentration of 40 mM (in 0.05% DMSO). Negative control incubations were treated only with phosphate-buffered saline for 1 h and then exposed for CCl_4_. Positive control incubations were incubated with Silymarin for 1 h before exposure to CCl_4_. All incubations continued for 24 h. Then, media and cell lysates were collected and stored at -20°C until analysis. Activities of alanine aminotransferase (ALT) and aspartate aminotransferase (AST) were assessed in the supernatant media. Glutathione (GSH) content and superoxide dismutase (SOD) activity were evaluated in the cell lysates using commercial kits (Biodiagnostics, Cairo, Egypt) according to the manufacturer’s instructions.

### Fractionation of the aqueous extract

Four hundred grams of the AE were suspended in 600 ml distilled water and fractionated over Diaion HP 20 column (60 cm L × 5 cm D, 500g) using 100% H_2_O, 50% MeOH in H_2_O, 100% MeOH, and (CH_3_)_2_CO yielding four fractions I-IV, respectively. The solvent in each case was evaporated under reduced pressure at 40°C to yield 160.47, 100.43, 8.20, and 1.56 g, respectively.

#### Isolation from the AE bioactive fractions (Fr I, II and IV)

Fr-I (10 g) was subjected to fractionation over a silica gel column (2 x 25 cm, 150 g). Gradient elution was performed using methanol-ethyl acetate mixtures. Fractions (100 ml, each) were collected and monitored by TLC using solvent system (**S**_**1**_). Sub-fraction (20% methanol in ethyl acetate) was chromatographed over a sephadex LH 20 column. The elution was carried out using 100% methanol to give two pure compounds **C1** (white amorphous powder, 322.5 mg) and **C2** (white amorphous powder, 278 mg).

Fr-II (20 g) was subjected to fractionation over a silica gel column (3.5 x 25 cm, 300 g). Gradient elution was performed using *n*-hexane-ethyl acetate mixtures. The polarity was increased by 1% every 100 ml till 100% ethyl acetate then ethyl acetate-methanol mixtures were used for elution by increasing polarity by 1% every 100 ml till 25% methanol. Fractions (100 ml, each) were collected and monitored by TLC using solvent system (S_1_). Sub-fractions (50% and 70% ethyl acetate in *n*-hexane) were separetly chromatographed over a sephadex LH 20 columns using (100% and 80% methanol, respectively) to yield pure compounds **C3** (white amorphous powder, 435.4 mg) and **C4** (yellow amorphous powder, 387.3 mg). Sub-fraction (5–10% methanol in ethyl acetate) was chromatographed over a sephadex LH 20 column. The elution carried out using 90% methanol to give compounds **C5** (yellow amorphous powder, 110.2 mg) and **C6** (white amorphous powder, 405.1 mg).

A weighed amount (1 g) of Fr-IV was subjected to fractionation over a silica gel column (1.5 x 20, 50g). Gradient elution was performed using methanol-dichloromethane mixtures. Fractions (100 ml, each) were collected and monitored by TLC using solvent systems (**S**_**2**_**-S**_**3**_). Sub-fraction (30–40% methanol in dichloromethane) was chromatographed over a silica gel column. The elution was carried out using dichloromethane–methanol (9.8:0.2 v/v). Similar fractions, showing pure spots, were pooled together to yield one pure compound **C7** (yellow amorphous powder, 264.8 mg).

### Evaluation of antioxidant activity

#### DPPH radical scavenging method

DPPH free radical scavenging activity of the aqueous plant extract was carried out using the method described by Shimada *et al*. [25]. Briefly, 0.1mM solution of DPPH^•^ in methanol was prepared. Then, 1 ml of this solution was added to 3 ml of extract solution at different conc. (25–75μg/ ml). The mixture was shaken vigorously and allowed to stand at room temperature for 30 min. Then the absorbance was measured at 517 nm in Asys microplate reader. Lower absorbance of the reaction mixture indicated a higher free radical scavenging activity.

DPPH scavenging effect (%) = 100 − [((A_0_-A_1_)/A_0_) × 100]

#### Ferric Reducing Antioxidant Power Assay (FRAP)

A slightly modified method of Benzei and Strain [26] was carried out to estimate the ferric reducing ability of the AE. Briefly, 10 μL of samples (AE and gallic acid) were mixed with 190 μL of reaction mixture (152 μL FRAP acetate buffer, pH 3.6, 19 μL ferric tripyridyl triazine (Fe III TPTZ) and 19 μL FeCl_3_). The absorbance was measured immediately at 594 nm in kinetic mode for 60 minutes at 37°C. To test the reproducibility of the assays, the antioxidant activities were measured three times. FRAP values (mM Fe (II)) were calculated using a linear regression equation for standard ferrous sulphate.

### *In vivo* experiments

#### Animals and experimental protocol

Male Wistar rats (200–250 g) were supplied by the Animal Breeding Laboratory, Helwan, Egypt. Animals were housed in an air-conditioned atmosphere (22±2°C, 12 h light–dark cycle). They were provided with rodent chow and water ad libitum. The investigation was performed in agreement with the Animal Research: Reporting of *in vivo* Experiments (ARRIVE) rules, created by the National Center for the Replacement, Refinement and Reduction of Animals in Research (NC3Rs). The investigation was approved by the Ethics Committee for Animal Experimentation of Faculty of Pharmacy, Cairo University (Permit Number: MP 2162).

#### LD_50_

LD_50_ was determined using acute toxic class method as per OECD (2001) (Organization for Economic Co-operation and Development, Guideline-423, adopted on 17^th^ December, 2001). Based on a previous pilot study in our laboratories, a limit test was performed. Animals were fasted overnight and the *P*. *niruri* was administered orally using gastric feeding needle at a dose of 5000 mg/kg (10 ml/kg dosing volume).

#### Hepatoprotective activity

To determine the least effective hepatoprotective dose, a dose-response study was conducted. Animals were divided into six groups of 6 rats each. Group I: control that received water via oral injection followed by intraperitoneal (IP) injection of corn oil after 4 h. Group II: CCl_4_ hepatotoxicity group which was injected once with 1 mL/kg of CCl_4_-corn oil 50% mixture. Groups III-VI: pretreated by oral bolus injection of AE (25, 50, 100 and 200 mg/kg, respectively), then given CCl_4_ IP injection after 4 h of the pretreatment. Rats were monitored twice a day to avoid any potential distress to animals. Signs of severe distress, abnormal behavior, writhing or convulsions were taken as indicators for immediate sacrifice of animals. However, none of such signs were observed with the doses used in the current study. Euthanasia was performed at 24 h after CCl_4_ challenge. At the end of the experiment, the rats were sacrificed under sodium pentobarbitone anaesthesia according to the guidelines for euthanasia in the Guide for the Care and Use of Laboratory Animals (2011). Histopathological findings together with AST and ALT levels were used to determine the least effective hepatoprotective dose to be used for further investigations.

#### Tissue and serum samples preparation

Blood samples were obtained from the retro-orbital plexus and allowed to clot. This was followed by centrifugation (3000 rpm for 10 min) to separate the serum. It was stored till needed at -80°C. Animals were then sacrificed and liver tissues were dissected and homogenized to produce a 20% homogenate.

#### Biochemical analyses

Activities of serum ALT, AST and alkaline phosphatase (ALP), serum levels of total bilirubin (TB), total proteins (TP), triglycerides (TG), total cholesterol (TC), creatinine, glucose, and urea were colorimetrically determined using kits (Spectrum Diagnostics, Cairo, Egypt). Lactate dehydrogenase (LDH) leakage was kinetically estimated using a commercial kit (BioSystems, Barcelona, Spain).

Thiobarbituric acid reactive substances (TBARS) level was measured as malondialdehyde (MDA) was estimated to determine the lipid peroxidation [27]. The reaction mixture composed of 0.5 ml homogenate, 2.5 ml 20% trichloroacetic acid (TCA), and 1.0 ml 0.6% thiobarbituric acid (TBA) was heated for 20 min then cooled and 4 ml *n*-butanol was added. The absorbance was measured at 535 nm. MDA level was expressed as nmol of MDA/g wet tissue.

To determine reduced glutathione (GSH), the mixture (0.5 mL homogenate+ 0.5 ml 10% TCA) was centrifuged for 10 min at 3000 rpm. The resulting supernatant (0.2 mL) was added to (1.7 mL phosphate buffer + 0.1 mL Ellman's reagent) then the absorbance was recorded within 5 min at 412 nm [28]. The results were expressed as μmol of GSH/g wet tissue.

The antioxidant enzymes catalase (CAT), glutathione reductase (GR), superoxide dismutase (SOD), and glutathione peroxidase (GSP) levels were determined using kits (Biodiagnostics, Cairo, Egypt). A fluorometric assay was used to determine levels of ROS, such as O_2_^−∙^, ^∙^OH, and H_2_O_2_. Nonfluorescent DCFDA was oxidized to the highly fluorescent 2',7'-dichlorofluorescin (DCF) in the presence of esterases and ROS, including lipid peroxides [29]. For the assay, 50 μM DCFDA was added to liver homogenates for 250 μL of final volume. Changes in fluorescence intensity were measured on a fluorescence plate reader, GENios (Tecan Instrument, Salzburg, Austria), with excitation and emission wavelengths set at 485 and 530 nm, respectively.

Total NO content was estimated spectrophotometrically in the liver homogenate by measuring absorbance of the formed chromophoric azo derivative [30]. For protein precipitation, the homogenate was incubated for 48 h with absolute ethanol. Then the supernatant was incubated with vanadium trichloride. This was followed by the addition of Griess reagent. The mixture was incubated for 30 minutes at 37°C and absorbance was measured at 540 nm. Results were expressed as μmol/g wet tissue.

#### Immunohistochemical assays

Liver sections (4 μm) were cut, fixed (65°C oven for 1 hr) and placed in 60 mL of triology working solution (Cell Marque, CA-USA. Cat# 920p-06). Sections were autoclaved and immersed in tris buffered saline. Three drops of rabbit polyclonal anti-rat cyclooxygenase-2 antibody (Thermoscientific, COX-2 Cat#RB-9072-R7) and/or rabbit polyclonal antibody to rat inducible nitric oxide synthase (Thermoscientific, iNOS Cat#RB-9242-R7) were applied then the slides were incubated. Biotinylated secondary antibody was added and incubated with the enzyme conjugate. Diaminobenzidine chromogen was added and rinsed. Light microscope examination after counterstaining was performed.

#### Protein level of inflammatory Cytokines (using ELISA)

Liver tissues from each group were homogenized in potassium phosphate buffer. The homogenates were centrifuged at 4000 xg for 10 min at 4°C. The supernatant was used for determination of the inflammatory cytokines. The levels of tumor necrosis factor-α (TNF-α), IL6, IL8, IL10 and NF-kB in tissue homogenates were determined by enzyme-linked immunosorbent assay (ELISA) using a rat immunoassay kit (RayBiotech, Norcross, USA) according to the recommendations of the manufacturer using a Microtiter plate reader capable of reading at 450 nm (Sunrise, Austria).

#### Histopathological study

Liver specimens from the two lobes were taken and fixed in 10% formalin then embedded in paraffin. Sections (4 μm thickness) were cut and stained with hematoxylin and eosin then examined under light microscopy.

### Statistical analysis

Results are presented as mean ± SD. Statistical analyses were carried out using One-way ANOVA followed by Tukey–Kramer as a post hoc test to conduct multiple comparisons for assessment of response variation among different groups. Differences between means were considered significant at p<0.05. The analyses were performed using GraphPad Instat software, version 3.

## Results

### *In vitro* hepatoprotective guided isolation of the major constituents

The results indicated that CCl_4_ induced toxicity in clone-9 cell line Table 1 and Table 2, and Hepg2 cell line Table 3 and Table 4, caused significant elevation of AST, ALT, GSH, and SOD levels. The toxicity induced by CCl_4_ in the clone-9 and Hepg2 cells was significantly (*p*< 0.001) recovered by treatment with *P*. *niruri* extracts. AE in all tested doses (0.01–1 mg/mL) significantly prevented CCl_4_ induced elevation of AST, ALT, GSH, and SOD levels. Its effect on these enzymes levels at 1 mg/ml approached that of the standard silymarin at the same dose (Table 1 and Table 3). It is worth to note that 50, 70 and 80% ethanol extracts of *P*. *niruri* had no significant amelioration to CCl_4_ toxicity.

**Table 1:** Protective effect of different extracts and fractions of *P*. *niruri* on CCl_4_ induced toxicity in clone-9 cell line.

Group	Dose mg/ml	AST U/mL	ALT U/mL	GSH mg/dL	SOD U/mL
**Control**		48.31±12.11	30.48±8.69	19.55±11.10	13.95±2.04
**CCl**_**4**_	**40mM**	124.06±6.84	73.22±5.12	66.09±3.57	38.87±7.33
**Silymarin**	**1**	50.01±3.25*	30.96±5.60*	21.26±8.00*	14.16±6.32
	**0.1**	61.33±1.22*	36.87±2.31*	26.77±2.21*	20.39±5.00
	**0.01**	77.02±1.02*	40.52±0.69*	33.28±1.65*	21.44±3.33
**AE**	**1**	81.03±5.62*	44.25±6.34*	33.57±7.26*	22.98±4.06
	**0.1**	82.11±2.36*	48.25±2.22*	36.28±1.26*	24.56±6.12
	**0.01**	89.43±6.25*	53.36±6.00*	36.89±4.53*	28.39±8.31
**Fr-I**	**1**	78.63±9.12*	42.54±3.25*	35.16±5.11*	23.15±4.12
	**0.1**	79.66±5.32*	44.36±4.65*	36.87±2.26*	25.01±3.72
	**0.01**	80.40±6.24*	45.03±5.08*	38.13±4.03*	26.54±2.29
**Fr-II**	**1**	77.12±1.63*	40.98±5.31*	30.95±4.06*	19.72±6.32
	**0.1**	77.73±1.26*	40.99±3.26*	29.84±6.28*	20.01±4.23
	**0.01**	78.01±1.01*	41.22±8.36*	30.15±9.14	20.98±4.69
**Fr-III**	**1**	99.86±9.11	60.52±5.59	66.35±4.53	46.38±6.66
	**0.1**	105.12±4.69	60.96±11.23	66.53±9.30	48.03±7.96
	**0.01**	112.25±10.32	62.53±8.32	70.05±9.33	50.36±10.23
**Fr-IV**	**1**	60.68±4.35*	41.35±8.32*	43.52±6.87*	18.67±7.62
	**0.1**	73.54±8.99*	41.80±12.36*	43.62±8.89*	26.54±8.71
	**0.01**	80.65±4.69*	44.63±8.79*	44.87±7.65*	29.56±4.09
**50% EtOH extract**	**1**	89.43±11.12	53.28±0.97	42.36±8.02	29.65±9.32
	**0.1**	95.87±2.36	64.32±11.30	47.99±4.33	33.57±2.19
	**0.01**	111.52±5.62	69.25±6.55	61.23±7.32	35.26±2.22
**70% EtOH extract**	**1**	90.03±10.36	50.27±3.02	49.26±15.03	28.63±7.64
	**0.1**	115.23±2.36	65.23±5.66	55.32±10.23	30.28±7.36
	**0.01**	118.23±3.65	69.52±4.86	61.53±8.01	32.21±6.86
**80% EtOH extract**	**1**	76.25±8.66	38.15±6.47	37.99±6.52	20.36±4.65
	**0.1**	80.95±6.57	42.65±9.35	43.21±6.23	25.36±4.69
	**0.01**	98.63±7.32	50.36±9.82	54.21±2.35	31.57±6.04

Values are mean±S.D. of three independent experiments carried out in triplicates.

*: Significance level at p< 0.001, compared to control and CCl_4_ groups; AE: aqueous extract.

**Table 2:** Protective effect of the isolated compounds from *P*. *niruri* on CCl_4_ induced toxicity in clone-9 cell line.

Group	Dose mg/ml	AST U/mL	ALT U/mL	GSH mg/dL	SOD U/mL
**Control**		62.35±5.32	34.63±0.36	36.75±3.58	22.97±5.16
**CCl**_**4**_	**40mM**	136.11±4.69	87.62±8.95	70.12±5.66	62.38±5.92
**Silymarin**	**1**	66.25±3.69*	36.52±11.56*	36.98±12.35*	25.59±4.21
	**0.1**	73.54±0.36*	40.58±13.51*	39.33±15.32*	28.28±6.21
	**0.01**	81.23±1.98*	48.62±13.25*	43.22±11.18*	33.26±6.23
**C1**	**1**	64.52±5.6*	35.68±17.23*	39.87±16.22*	23.01±3.36
	**0.1**	76.58±6.58*	43.56±20.00*	43.68±18.30*	26.71±5.06
	**0.01**	82.59±6.08*	50.06±16.33*	49.62±16.35*	29.63±7.31
**C2**	**1**	69.52±2.06*	40.25±18.62*	40.32±10.62*	27.59±5.12
	**0.1**	83.26±7.12*	46.52±16.52*	49.61±13.49*	31.26±4.26
	**0.01**	91.03±2.03*	50.68±19.24*	53.22±16.15*	33.68±1.15
**C3**	**1**	95.24±2.33*	58.21±22.30*	49.55±17.98*	34.52±2.05
	**0.1**	100.25±3.02*	65.31±22.37*	54.29±22.21	39.62±5.21
	**0.01**	111.03±5.03*	70.36±24.03*	58.09±30.04*	43.34±6.04
**C4**	**1**	106.25±5.03*	54.32±22.05*	50.62±19.32*	40.68±6.07
	**0.1**	112.87±1.03*	55.06±23.28*	50.01±25.03*	41.11±0.36
	**0.01**	120.71±1.06*	56.87±16.24*	57.62±14.21*	50.28±3.68
**C5**	**1**	70.36±6.24*	41.62±19.22*	39.99±20.17*	27.35±5.17
	**0.1**	75.68±5.01*	43.05±11.56*	40.98±18.42*	28.95±1.03
	**0.01**	81.24±2.04*	50.32±10.57*	44.68±17.62*	30.39±9.24
**C6**	**1**	99.04±0.28*	52.16±9.08*	49.27±16.27*	40.16±1.19
	**0.1**	99.02±2.06*	51.97±11.67*	47.62±24.26*	41.03±5.16
	**0.01**	106.52±5.17*	55.67±19.07*	56.27±18.09	41.36±2.58
**C7**	**1**	69.51±8.31*	43.28±19.20*	38.62±22.51*	26.24±1.16
	**0.1**	81.62±5.07*	49.51±9.27*	43.26±15.20*	30.25±7.16
	**0.01**	97.57±1.67*	58.62±16.27*	48.62±11.09*	37.82±2.08

Values are mean±S.D. of three independent experiments carried out in triplicates.

*: Significance level at p< 0.001, compared to control and CCl_4_ groups; C1: corilagin; C2: isocorilagin; C3: brevifolin; C4: quercetin; C5: kaempferol rhamnoside; C6: gallic acid; C7: brevifolin carboxylic acid

**Table 3:** Protective effect of different extracts and fractions of *P*. *niruri* on CCl_4_ induced toxicity in Hepg2 cell line.

Group	Dose	AST	ALT	GSH	SOD
	(mg/mL)	(U/mL)	(U/mL)	(mg/dL)	(U/mL)
**Control**		51.32±6.28	28.80±3.85	21.45±15.45	18.55±3.00
**CCl**_**4**_	40 mM	116.55±9.61	63.09±7.61	48.12±3.30	43.30±5.50
**Silymarin**	**1**	55.11±2.03*	28.58±3.54*	22.44±8.93*	21.15±1.93*
	**0.1**	59.46±0.67*	30.07±4.58*	24.01±2.25*	23.24±1.74*
	**0.01**	67.49±1.77*	34.63±4.47*	27.39±2.57*	26.10±2.19*
**AE**	**1**	76.93±7.57*	42.25±4.05*	31.92±2.03*	28.27±3.98*
	**0.1**	77.32±3.70*	40.51±5.06*	31.58±6.12*	29.44±2.92*
	**0.01**	79.95±3.80*	41.88±5.85*	32.65±7.02*	30.45±3.01*
**Fr-I**	**1**	73.82±3.12*	38.47±4.99*	30.10±2.18*	28.22±2.68*
	**0.1**	76.35±1.54*	38.95±5.90*	30.92±2.91*	29.65±2.37*
	**0.01**	78.88±2.48*	40.68±5.02*	32.06±7.32*	30.39±2.66*
**Fr-II**	**1**	64.70±96.97*	35.83±4.82*	26.92±9.92*	23.62±3.52
	**0.1**	65.06±3.40*	34.23±4.60*	26.61±1.82*	24.69±2.53
	**0.01**	67.48±3.50*	35.49±4.24*	27.59±2.65	25.62±2.62
**Fr-III**	**1**	104.69±8.95	56.82±6.70	43.26±3.35	38.81±5.03
	**0.1**	105.15±4.37	54.76±7.26	42.86±5.86	40.21±3.80
	**0.01**	108.25±4.49	56.37±7.37	44.13±3.92	41.40±3.91
**Fr-IV**	**1**	57.79±11.54*	34.66±3.71*	34.66±2.71*	19.84±4.61*
	**0.1**	64.35±3.84*	34.09±4.78*	26.38±2.34*	24.29±2.63*
	**0.01**	79.17±7.46*	43.32±5.71*	32.80±4.89*	29.17±4.00*
**50% EtOH extract**	**1**	87.56±4.50	46.03±5.73	35.80±9.41	33.25±3.39
	**0.1**	93.43±5.23	49.33±6.36	38.26±3.15	35.36±3.73
	**0.01**	101.13±3.49	52.31±6.04	41.13±4.86	38.87±3.48
**70% EtOH extract**	**1**	100.90±3.63	52.27±6.77	41.06±4.70	38.74±3.51
	**0.1**	101.59±2.49	52.04±7.07	41.19±3.57	39.33±3.26
	**0.01**	105.724±1.72	53.72±7.54	42.76±7.49	41.17±3.20
**80% EtOH extract**	**1**	81.021±4.32	42.67±5.26	33.15±2.13	30.73±3.18
	**0.1**	86.65±5.02	45.84±5.72	35.50±8.81	32.75±3.51
	**0.01**	94.04±3.35	48.70±6.13	38.27±2.37	36.12±3.26

Values are mean±S.D. of three independent experiments carried out in triplicates.

*: Significance level at p< 0.001, compared to control and CCl_4_ groups; AE: aqueous extract.

**Table 4:** Protective effect of the isolated compounds from *P*. *niruri* on CCl_4_ induced toxicity in Hepg2 cell line.

Group	Dose	AST	ALT	GSH	SOD
	(mg/mL)	(U/mL)	(U/mL)	(mg/dL)	(U/mL)
**Control**		51.32±6.28	28.80±3.85	21.45±15.45	18.55±3.00
**CCl**_**4**_		116.55±9.61	63.09±7.61	48.12±3.30	43.30±5.50
**Silymarin**	**1**	55.11±2.03*	28.58±7.54*	22.44±8.93*	21.15±1.93*
	**0.1**	59.46±0.67*	30.07±8.58*	24.01±11.25*	23.24±1.74*
	**0.01**	67.49±1.77*	34.63±6.47*	27.39±9.57*	26.10±2.19*
**C1**	**1**	56.08±3.66*	29.87±13.07*	23.03±8.44*	21.09±2.38*
	**0.1**	65.72±3.49*	34.59±14.04*	26.88±12.04*	24.94±2.56*
	**0.01**	73.77±3.69*	38.73±15.56*	30.15±14.83*	28.04±2.83*
**C2**	**1**	62.28±3.98*	33.13±13.22*	25.56±9.52*	23.44±2.62*
	**0.1**	72.85±2.79*	37.82±14.54*	29.67±11.97*	27.93±2.57*
	**0.01**	78.94±5.37*	42.16±12.03*	32.45±12.84*	29.63±3.41*
**C3**	**1**	73.28±4.11*	38.69±14.91*	30.01±14.44*	27.74±2.93*
	**0.1**	78.63±4.77*	41.70±12.22*	32.255±12.03*	29.66±3.24*
	**0.01**	85.66±3.18*	44.42±18.32*	34.87±12.41*	32.86±3.00*
**C4**	**1**	85.45±3.32*	44.38±18.08*	34.80±12.26*	32.74±3.03*
	**0.1**	86.08±2.27*	44.17±15.26*	34.92±10.05*	33.28±2.80*
	**0.01**	89.86±1.57*	45.71±16.42*	36.36±13.80*	34.96±2.74*
**C5**	**1**	59.75±3.81*	31.78±13.55*	24.53±9.68*	22.49±2.51*
	**0.1**	70.09±2.79*	36.44±11.59*	28.56±11.97*	26.84±2.50*
	**0.01**	76.07±5.21*	40.64±8.11*	31.27±12.88*	28.54±3.29*
**C6**	**1**	80.60±3.22*	41.91±14.72*	32.84±12.55*	30.86±2.88*
	**0.1**	81.22±2.20*	41.71±15.87*	32.96±12.32*	31.38±2.65*
	**0.01**	84.88±1.53*	43.20±18.94*	34.345±13.02*	33.01±2.59*
**C7**	**1**	58.48±3.65*	31.07±13.77*	23.10±15.31*	22.04±2.44*
	**0.1**	68.48±3.49*	35.97±14.99*	27.99±13.04*	26.03±2.63*
	**0.01**	76.53±3.69*	40.11±11.51*	31.26±12.83*	29.13±2.90*

Values are mean±S.D. of three independent experiments carried out in triplicates.

*: Significance level at p< 0.001, compared to control and CCl_4_ groups; C1: corilagin; C2: isocorilagin; C3: brevifolin; C4: quercetin; C5: kaempferol rhamnoside; C6: gallic acid; C7: brevifolin carboxylic acid.

Fractionation of AE on Diaion HP20 yielded four subfractions I-IV, on testing their *in vitro* hepatorotective activity on clone-9 and Hepg2 cell lines, Fr I, II, and IV (0.01–1 mg/ml) significantly (*p*< 0.001) reduced CCl_4_ induced elevation of AST, ALT, and GSH. Fr I and IV also caused significant (*p*< 0.001) reduction in SOD levels at all tested doses. Fr-IV was the most active fraction in reduction of all toxicity parameters. Treatment of Hepg2 cells with 1 mg/ml Fr-IV reduced AST, ALT, GSH, and SOD levels to 57.79 ± 11.54, 34.66 ± 3.71, 34.66 ± 2.71, and 19.84 ± 4.61 respectively. It reduced SOD more than the reduction caused by silymarin (21.15 ± 1.93) at a dose of 1mg/mL.

**Fig 1:**
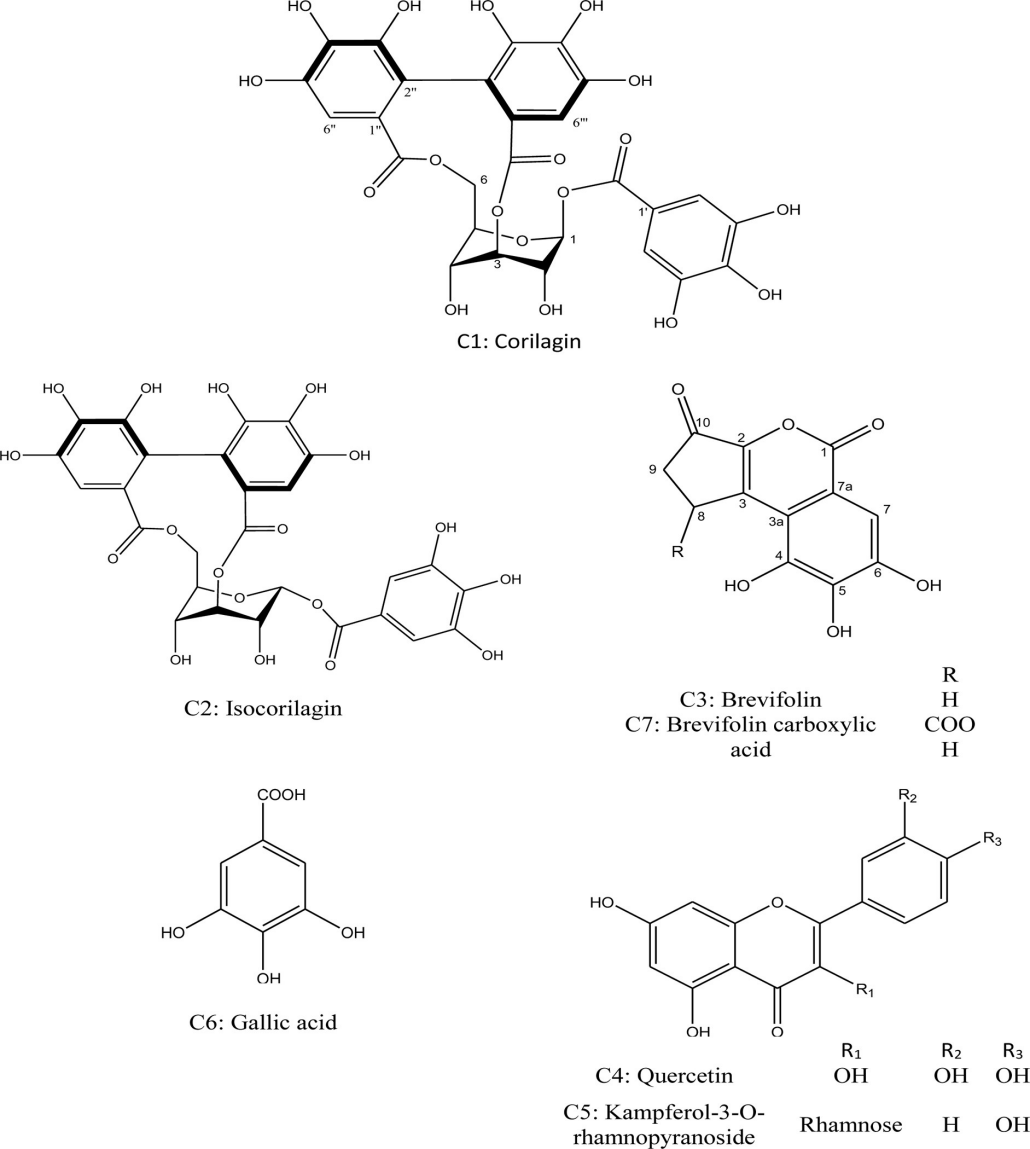
Chemical structures of the compounds isolated from *P*. *niruri*.

Purification of fractions I, II and IV yielded seven compounds corilagin (*β*-1-*O*-galloyl-3, 6-(*R*)-hexahydroxydiphenoyl-D-glucose) **C1,** isocorilagin (*α*-1-*O*-galloyl-3, 6-(*R*)-hexahydroxydiphenoyl-D-glucose) **C2** (Sprenger *et al*., 2016), brevifolin **C3** [31], quercetin **C4**, kaempferol rhamnoside **C5** [32], gallic acid **C6** and brevifolin carboxylic acid **C7** [33]. **C1-7** structures were established on the basis of physicochemical properties and spectral analysis (^1^H, ^13^C-NMR, COSY, HSQC, and HMBC). ^1^H and ^13^C-NMR are shown in S1 and S2 Tables and the structures of the compounds are presented in Fig 1.

Surprisingly, all isolated compounds (**C1-C7)** at different tested doses 0.01–1 mg/ml significantly (p< 0.001) reduce CCl_4_ induced elevation of AST, ALT. They also restore the activity of SOD and GSH in clone-9 and Hepg2 cell lines. Corilagin **C1**, isocorilagin **C2**, kaempferol rhamnoside **C5**, and brevifolin carboxylic acid **C7** demonstrated strong hepatoprotective activity, whereas, brevifolin **C3**, quercetin **C4** and gallic acid **C6** showed moderate activity.

### Antioxidant activity

#### DPPH radical scavenging method

The AE showed a better antioxidant potential when compared to standard ascorbic acid. IC 50 obtained values were 11.6 ± 2 and 12± 3.5 μg/ml. for AE and ascorbic acid respectively. It means that AE, at a higher concentration, captured more free radicals formed by DPPH resulting in increasing IC 50 value.

#### Ferric Reducing Antioxidant Power Assay (FRAP)

Tested AE and gallic acid showed FRAP values of 79.352 ± 2.88 and 62.85±4.08 mM Ferrous equivalents, respectively. Results showed that the AE has a higher FRAP value than gallic acid; indicating more electron donating capacity of AE in comparison to the standard gallic acid.

### *In vivo* hepatoprotective activity of AE

#### LD_50_ study

No mortality was observed after oral administration of AE up to 5000 mg/kg, hence, the extract is considered GHS category 5 or unclassified with LD_50_ cut off greater than 5000 mg/kg. Also, no food aversion or adverse behavioral changes was observed in treated rats.

#### Dose-response and histopathological findings

**Fig 2:**
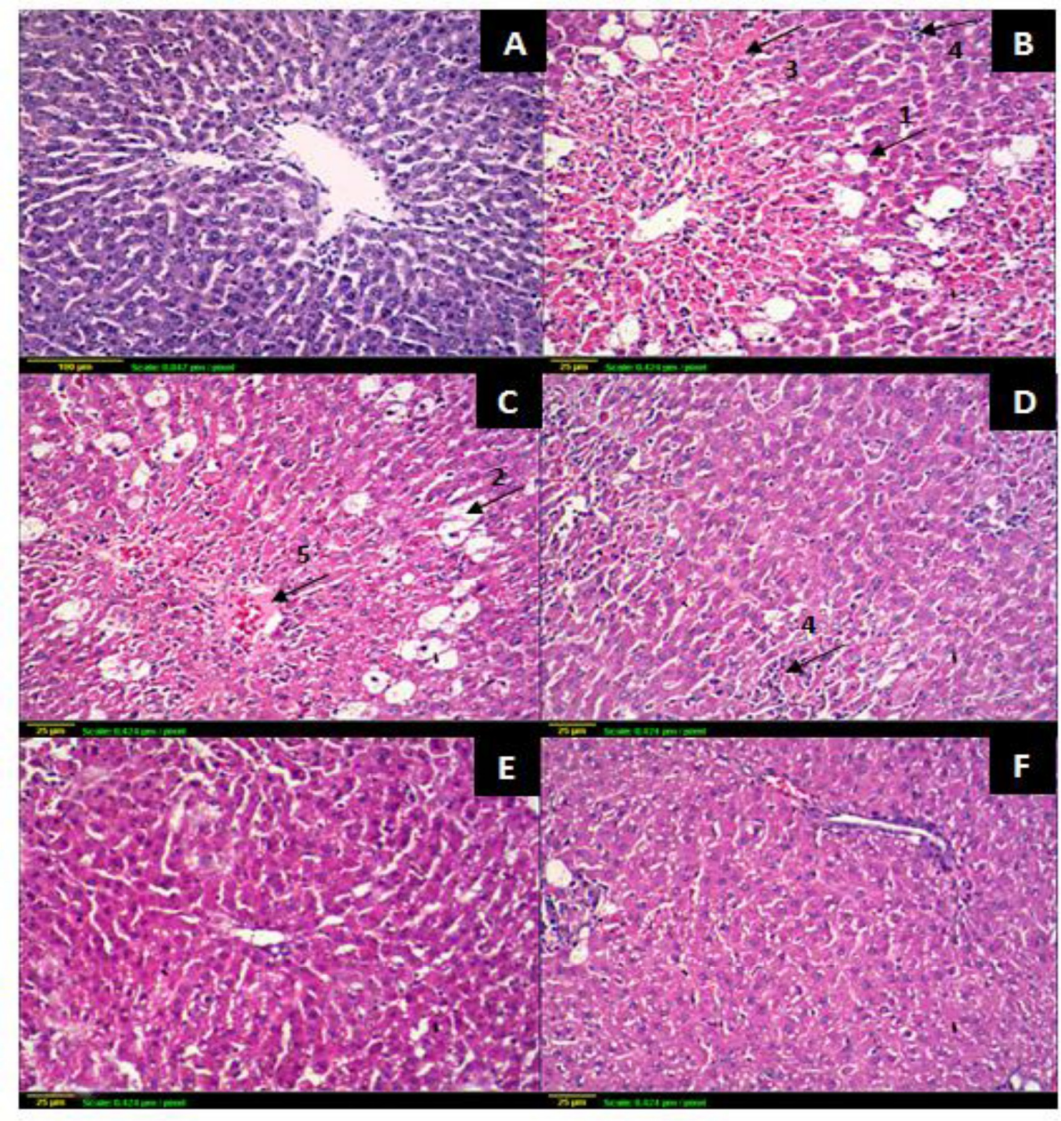
Effect of *P*. *niruri* AE on rats liver histopathology after acute CCl_4_ intoxication. **(A)** control portal area and surrounding hepatocytes; **(B)** CCl_4_; **(C)** CCl_4_ + 25 mg AE; **(D)** CCl_4_+ 50 mg AE; (**E)** CCl_4_+ 100 mg AE; **(F**) CCl_4_+200 mg AE. (x100), n = 6. (1): severe ballooning degeneration; (2): fatty changes; (3): necrosis; (4): inflammatory cells infiltration; (5): marked congestion.

To determine the least effective hepatoprotective dose of AE, AST and ALT levels were determined after CCl_4_ challenge in rats pretreated with AE. Histopathological findings are presented in Table 5 and Fig 2. A 2.5 and 6 fold increase of AST and ALT levels, respectively, in CCl_4_ group compared to the control group was observed. The 25 and 50 mg kg^-1^day^-1^ doses were unable to produce significant reversal of these enzymes. However, a significant decrease was observed starting from a dose of the 100 mg kg^-1^day^-1^.

**Table 5:** Effect of *P*. *niruri* AE different doses on AST and ALT levels after rat acute CCl_4_ intoxication.

Group	AST (U/L)	ALT (U/L)
**Control**	134.21±33.49***	40.19±10.11***
**CCl**_**4**_ **(1 mL/kg)**	327.19±28.04	247.65±17.59
**CCl**_**4**_**+ AE (25 mg/kg)**	320.63±45.51	233.7±20.38
**CCl**_**4**_**+ AE (50 mg/kg)**	314.91±32.87	228.15±14.93
**CCl**_**4**_**+ AE (100 mg/kg)**	252.98±5.71**	183.43±47.1**
**CCl**_**4**_**+ AE (200 mg/kg)**	231.93±34.67***	176.25±38.1**

Values are mean± SD.

*: *p*<0.05

**: *p*<0.01

***: *p*<0.001 compared to CCl_4_ group. AE: aqueous extract.

The histological observations shown in Fig 2 supported these results where a dose-dependent restoration of cellular integrity was confirmed. The CCl_4_ group showed a marked destruction of the hepatic architecture, accompanied with severe fatty changes, severe ballooning degeneration, necrosis, and inflammatory cells infiltration. A dose of 25 mg kg^-1^day^-1^ of AE with CCl_4_ causes a similar damage with a marked congestion, while a dose of 50 mg kg^-1^day^-1^ of AE demonstrates a lesser damage with a marked inflammation. Administration of AE (100 & 200 mg kg^-1^day^-1^) showed a marked improvement with restoration of cellular architecture. Thus, the least dose of AE that showed hepatic architecture protection is 100 mg kg^-1^day^-1,^ therefore it was used for all the other investigations.

#### Hepatotoxicity and oxidative stress markers

Effect of AE (100 mg kg^-1^day^-1^, p.o.) on hepatic toxicity indices, oxidative stress markers, and antioxidant enzymes in rats subjected to acute CCl_4_ intoxication are summarized in Table 6.

**Table 6:** Effect of *P*. *niruri* AE (100 mg kg^-1^day^-1^) on hepatic toxicity indices, oxidative stress markers, inflammatory markers and antioxidant enzymes after rats acute CCl_4_ intoxication.

		Control	CCl_4_	CCl_4_+ AE
**H.T.I.**	**LDH (U/L)**	173.58±64.56***	408.16±40.99	258.44±58.38***
	**ALP (U/L)**	153.8±31.27**	239.4±47.4	176.8±29.44*
	**TB (mg/dl)**	0.24±0.0077*	0.26±0.0061	0.25±0.01*
	**TP (g/dl)**	7.02±1.36*	4.88±0.73	6.11±1.23*
	**TC (mg/dl)**	47.42±8.56*	77.55±28.54	45.35±10.75*
	**TG (mg/dl)**	40.66±15.56**	87±34.75	33.7±7.44**
	**Glucose (mg/dl)**	190.64±40.74	153.75±12.12	190.26±42.45*
	**Urea (mg/dl)**	29.08±10.12*	39.57±2.86	29.51±5.5*
	**Creatinine (mg/dl)**	2.45±0.27***	4.09±0.21	3.75±0.15*
**O.S.M.**	**MDA (nmol/g)**	35.17±3.31***	60.11±8.09	38.03±2.88***
	**GSH (μmol/g)**	3.53±0.92**	1.997±0.49	3.54±0.83**
	**ROS (Flu/min/mg protein)**	5.2±0.25**	7.8±0.31	5.7±0.33**
**I.M.**	**NF-kB (ng/mg protein)**	15.5 ± 0.15	32.5 ± 0.23***	24.3 ± 0.18**
	**IL-6 (ng/mg protein)**	45.3 ± 3.19*	11.8 ± 4.21	49 ± 4.65*
	**IL-8 (ng/mg protein)**	55 ± 11.42	98 ± 15.32 ***	65 ± 8.25
	**IL-10 (ng/mg protein)**	35 ± 8.52	66 ± 6.5***	51 ± 9.32**
	**TNF (ng/mg protein)**	21 ±8.1*	32 ± 3.6	23 ± 2.9*
**A.O.E.**	**SOD (U/mg wet tissue)**	68.75±10.46***	217.42±81.34	106.25±34.69**
	**CAT (U/mg wet tissue)**	160.97±18.43*	180.71±8.46	163.43±9.06*
	**GSP (U/mg wet tissue)**	17.66±3.05	14.73±3	20.29±6.68*
	**GR (U/mg wet tissue)**	15.71±1.27*	12.4±2.39	15.68±1.3*

Values are mean ± SD. n = 6.

*: *p*<0.05

**: *p*<0.01

***: *p*<0.001 compared to CCl_4_ group. AE: aqueous extract; A.O.E: antioxidant enzymes; H.T.I: hepatic toxicity indices; I.M.: inflammatory markers; O.S.M: oxidative stress markers.

After the CCl_4_ challenge, significant changes were observed in the hepatic toxicity indices, there was an obvious LDH leakage evident through the 2.5 fold increase in its serum levels compared to the control group. Serum levels of ALP, TB, TC, TG, urea and creatinine significantly increased, too. Pretreatment of animals with AE significantly (*p*<0.05–0.01) reduced levels of theses hepatotoxicity markers compared to CCl_4_ group. Also a marked depletion of serum TP, accompanied by hypoglycemia was observed in CCl_4_ group. Although no statistically significant changes in glucose levels in the pretreated rats were recorded, the differences are easily observed. AE increased TP levels, however this increase did not reach that of the control. Glucose levels were normalized by administration of AE.

CCl_4_ increases MDA levels two folds of the control group and caused GSH depletion accompanied with 1.5 fold increase of ROS. These changes in the oxidative stress markers were successively restored by AE pretreatment. Pretreatment with AE decreased the MDA level to reach 38.03 ± 2.88 nmol/g, instead of being 60.11 ± 8.09 nmol/g in CCl_4_ group, this value is similar to some extent to that of the control group (35.17 ± 3.31 nmol/g). The extract was able to restore the GSH depletion caused by CCl_4_ to 3.54 ± 0.83 μmol/g which is equal to its level in the control group. Moreover, the ROS were diminished in AE treated group to show no significant difference when compared with the control group.

As regards the antioxidant enzymes activities, SOD and CAT levels, increased by the CCl_4_ administration and approached normal levels by AE pretreatment. On the other hand, GSP and GR activities were reduced by CCl_4_ challenge and boosted significantly by AE pre-administration. GSP and GR activities reach 20.29 ± 6.68 U/mg wet tissue and 15.68 ± 1.3 U/mg wet tissue respectively in the group pretreated with the extract. These values are higher than or even equal to their activities in the control group.

#### Inflammatory markers

**Fig 3:**
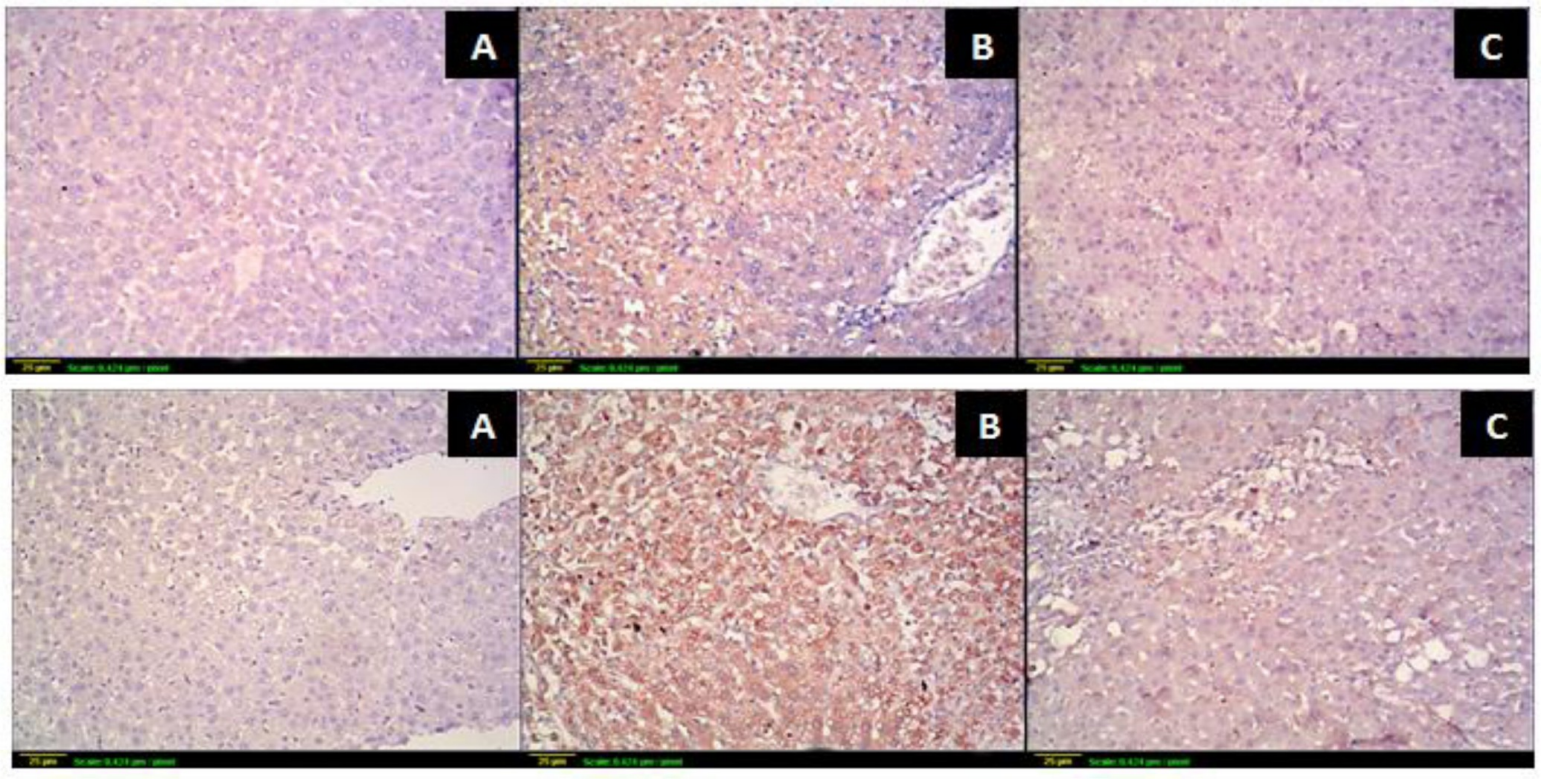
Immunohistochemical effect of *P*. *niruri* AE (100 mg kg^-1^day^-1^) on COX-2 and iNOS expression. Expression of COX-2 (Higher panel) and iNOS (lower pannel) by immunohistochemical staining (x100); **(A):** control; **(B)** CCl_4_; **(C)** CCl_4_+ 100 mg kg^-1^ day^-1^ *P*. *niruri* AE.

**Fig 4:**
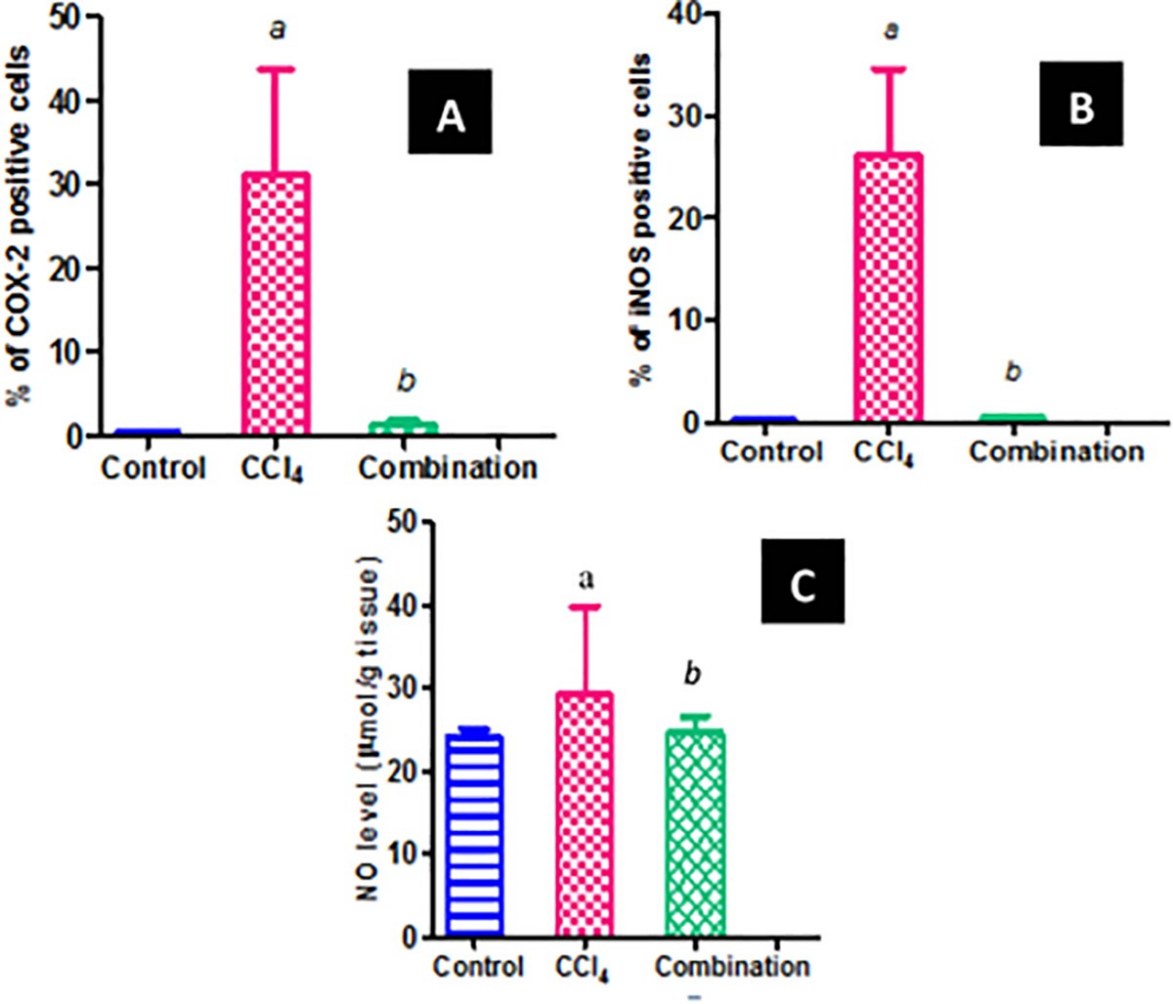
Effect of *P*. *niruri* AE (100 mg kg^-1^day^-1^) on COX-2 and iNOS. **(A)** % of COX-2; **(B)** % of iNOS positive cells as measured by image analysis; **(C)** NO levels; *a*: significantly different from control group; *b*: significantly different from CCl_4_ group.

Studying AE effect on inflammatory markers revealed potent activity. The immunohistochemical study revealed increase in hepatic COX-2 and iNOS expression (Figs 3 and 4) after CCl_4_ administration. CCl_4_ caused extensive expression appeared as intense brown color. Combination of CCl_4_ and AE (100 mg kg^-1^day^-1^, p.o.) caused the extent of expression to decrease markedly denoted by less brown color. In other words, AE significantly attenuated the increase of COX-2 and iNOS caused by CCl_4_. In addition, CCl_4_ challenge was accompanied by excessive production of the pro-inflammatory mediators NO and TNF-α. The NO elevation caused by CCl_4_ was attenuated by AE pretreatment and its level approached that of the control. Nevertheless, the higher amounts of TNF-α, NF-KB, IL-6, IL-8 and IL10, revealed by ELISA technique in the CCl_4_ group, were decreased by AE administration.

## Discussion

The plant-derived natural products have long been sources of drugs and medicinal agents. Their large diversity makes them very important in drug discovery, leading to the identification of novel molecules that have various physiological and pathological effects.

Hepatic diseases are a group of diseases which could be effectively prevented or treated by natural products. They constitute a serious global concern, having fatal complications which can end in death. Accordingly, finding a safe, effective, protective and several-mechanistic drug can provide an optimum solution against these complications.

In this study, the hepatoprotective effect of *P*. *niruri* was investigated using CCl_4_ induced hepatotoxicity in the clone-9 and Hepg2 cell lines.

*In vitro* cytotoxicity and hepatoprotective potential of plants using Hepg2 are important for primary screening, as it is an ideal model for studying *in vitro* xenobiotic metabolism and liver toxicity [34]. CCl_4_ caused the production of reactive oxygen species (ROS) in a time-dependent manner which is usually accompanied by lipid peroxidation which reaches its maximum after 24h of incubation. Thus, clone-9 and Hepg2 cells were incubated for 24 h with CCl_4_ for studying the hepatoprotective effect of AE against CCl_4_-induced toxicity [34].

The released leakage enzymes-AST and ALT indicate the extent of cellular damage, this release was observed at 24 h exposure to CCl_4_. The hepatotoxin also induces membrane damage and oxidative injury which leads to a significant decrease in SOD activity and GSH content in cells. Treatment of the cells with different concentrations of AE caused a significant restoration of the altered parameters towards the normal. Possibly the mechanism underlying AE potent hepatoprotective effect is its ability to maintain glutathione in the reduced state by virtue of its antioxidative powers and inhibit lipid peroxidation.

Fractionation of AE on Diaion HP20 yielded four subfractions I-IV, on testing their *in vitro* hepatoprotective activity on clone-9 and Hepg2 cell lines, only I, II and IV showed significant activity through reducing the levels of ALT, AST, the activity of SOD and GSH content.

Purification of fraction I yielded corilagin (*β*-1-*O*-galloyl-3,6-(*R*)-hexahydroxydiphenoyl-D-glucose) **C1** and isocorilagin (*α*-1-*O*-galloyl-3,6-(*R*)-hexahydroxydiphenoyl-D-glucose) **C2**, fraction II yielded brevifolin **C3**, quercetin **C4**, kaempferol rhamnoside **C5,** gallic acid **C6**, and fraction IV yielded brevifolin carboxylic acid **C7**.

^1^H- NMR spectral data (DMSO-*d*_*6*_) of compound **C1** showed a β glucose moiety assigned as 6.22 (d, *J* = 7.5 Hz, H-1), 3.89 (d, *J* = 7.5 Hz, H-2), 4.61 (br.s, H-3), 4.28 (br.s, H-4), 4.37 (t, *J* = 8.1 Hz, H-5), 4.24 (dd, *J* = 10.8, 7.7 Hz, H-6a), 3.97 (dd, *J* = 10.8, 10.2 Hz, H-6b), singlet at 7.03 ppm indicating the galloyl protons and two singlets appeared as 6.51 and 6.58 ppm assigned to the hexahydroxydiphenoyl protons.^13^C-NMR spectral data also showed the characteristic signals of the *β* glucose, galloyl moiety and the two hexahydroxydiphenoyl moieties. The HMBC correlations indicated that **C1** could be identified as corilagin. Compound **C2** was identical to **C1** in every respect except the doublet of the anomeric H at *J* = 2.2 Hz, indicating the α-configuration, thus compound **C2** was identified as isocorilagin [35].

The ^1^H-NMR spectra (DMSO-*d*_*6*_) of **C7** showed two aliphatic protons at δ_H_ 2.98 (2H, dd) and 4.42 (1H, dd) assigned to the methylene Hs at CH_2_-9 and the methine H at CH-8, respectively, in addition to a singlet from the aromatic proton at δ 7.29 ppm assigned to H-7.

The ^13^C NMR spectrum of **C7** exhibited 13 individual C signals, among which the most two upfield resonances at δ 37.8 and 42.8 ppm were attributed to the aliphatic methylenic C-9 and the methinic C-8, while the three most downfield signals at δ 161.5, 195.4 and 173.9 ppm were assigned to the carbonyl C-1, C-10, and COOH, respectively. This compound was identified as brevifolin carboxylic acid. The ^1^H-NMR and ^13^C-NMR spectra (DMSO-*d*_*6*_) of **C3** is similar to C7 except for the absence of the signal at δ_C_ 173.9 (COOH) with the subsequent upfield shift of C-9 and C-8 which appeared at δ_C_ 33.5 and 24.3 ppm, respectively. Thus, compound **C3** was identified as brevifolin.

Few reports were traced on the hepatoprotective and antioxidant activities of some of the proposed isolated compounds. Quercetin **C4** produced a significant hepatoprotection against country made liquor and paracetamol challenge models [36]. Gallic acid **C6** significantly reverses the increased levels of liver marker enzymes, TNF-α, lipid peroxidation levels, and the depleted antioxidant status in paracetamol-challenged mice [37]. Corilagin **C1** possesses antioxidant, hepatoprotective activities *via* scavenging action for O_2_^·−^ and peroxyl radicals and also inhibited ROS production from leukocytes stimulated by phorbol-12-myristate acetate [38]. It significantly reduced galactosamine (GalN) and lipopolysaccharide (LPS) induced hepatotoxicity. It reduced ALT, AST, and decreased radical formation and lipid peroxidation in GalN/LPS treatment model [38]. Nothing was traced on isocorilagin **C2**, brevifolin **C3,** kaempferol rhamnoside **C5**, and brevifolin carboxylic acid **C7**.

No data was traced on the hepatoprotective activity of the isolated compounds against CCl_4_-induced hepatotoxicity on the clone-9 and Hepg2 cell lines. Hence, the authors were encouraged to test the isolates on CCl_4_-induced hepatotoxicity in the clone-9 and Hepg2 cell lines. Compounds **C1-C7** at different tested doses reduced CCl_4_ induced elevation of AST, ALT, and restored the activity of SOD and GSH in the clone-9 and Hepg2 cell lines. Corilagin **C1**, isocorilagin **C2,** kaempferol rhamnoside **C5,** and brevifolin carboxylic acid **C7** demonstrated strong hepatoprotective activity. Whereas, brevifolin **C3**, quercetin **C4**, gallic acid **C6** showed moderate activity.

AE was chosen for the *in vivo* testing as the plant used in traditional medicine in Malaysia and other countries as a herbal tea and we needed to put a scientific basis for this traditional use. The present *in vivo* study presents the hepatoprotective actions of AE in an acute CCl_4_-induced hepatic injury model in rats. Liver injury induced by CCl_4_ is a common model for hepatoprotective effect screening, where a single exposure can rapidly lead to severe hepatic steatosis and necrosis. The 1-day model was implemented because CCl_4_ has the peak of its deleterious toxic effects at 24 h post-injection, then values were slowly normalized [39–41]. Thus, a drug like *P*. *niruri*, which can reverse CCl_4_ damage during its peak, would be a perfect candidate for treating liver diseases. In addition, the fact that the LD_50_ has not been yet reached by a dose of 17g/kg proves that the drug is highly safe, as according to Horn and Rhiouani [42, 43] who reported that plant extracts with LD_50_ values higher than 2–3 g/kg are considered nontoxic.

CCl_4_ causes drastic increases in serum levels of AST, ALT, ALP, LDH, TC, TG, total bilirubin, urea and creatinine. These effects can be attributed to the hepatocellular damage it causes during the course of its metabolism [44, 45]. This was evident in the histopathology slides where CCl_4_ caused a complete destruction of the cellular architecture. Pretreatment of rats with AE reduced these alterations successively. It is a remarkable fact that TC and TG were decreased by AE to levels below the control group, suggesting an antihyperlipidemic effect, a property presented in a previous study [22].

The observed effects of CCl_4_ on serum parameters as TC, TG, TP and glucose gain supported by previous studies [46]. Cholesterol efflux capacity is strongly associated with liver disease mortality [47]. The observed restoration of normal levels of TC and TG indicates compensated liver function supported by administration of AE. Also, this lipid lowering activity might be explained based on the findings of Khanna *et al*.[48] who reported that *Phyllanthus niruri* inhibits hepatic cholesterol biosynthesis, increases fecal bile acids excretion and enhances plasma lecithin.

The obtained data indicated that CCl_4_ intoxication was associated with hypoglycemia. This is consistent with the reported toxicity of the CCl_4_ to hepatocytes as it causes an energy deficit and a decline in glycogen content that ultimately leads to hypoglycemia [49]. Therefore, it can be concluded that the known anti-apoptotic properties of the *Phyllanthus* species [50] are involved in protecting against hepatocyte damage by CCl_4_. Also, the ability of the plant extract to mitigate CCl4-induced decline in serum TP highlights the ability of *Phyllanthus niruri* to improve the synthetic function of the liver. This is in line with previous reports [51].

Lipid peroxidation is perceived as one of the principal steps of CCl_4_-induced liver damage. Therefore, the antioxidant activity is a very important property in hepatoprotection. In this study, there was an increase in MDA with concomitant decrease in GSH, GSP, and GR after CCl_4_ challenge. However, their levels in the pretreated group were comparable to those of the control group, indicating that AE has a significant free radical scavenging antioxidant activity. MDA has long been used as a biomarker of oxidative stress [52, 53], and the increase of MDA reflects enhanced lipid peroxidation and tissue injury. GSH on the other hand, represents the non-enzymatic part of the host antioxidant defense mechanism, whereas SOD, CAT, GSP, and GR constitute the enzymatic part. GSH can effectively scavenge free radicals, where it is oxidized by GSP into glutathione disulfide which can be reduced back to GSH by GR with the consumption of NADPH [54]. GSH can react also with different electrophiles, xenobiotics and physiological metabolites to form mercapturates, which are catalyzed by other antioxidant enzymes. SOD mainly catalyzes the conversion of the highly reactive superoxide anion to O_2_ and to the less reactive species H_2_O_2_, which can be then destroyed by CAT and GSP [55].

As seen from the present study, these enzymes are functionally and structurally impaired by the overload of free radicals resulting in their dysfunction during hepatotoxicity [56]. However, SOD and CAT activities were increased by CCl_4_, in contrast to GSP and GR. Several studies have found a decreased antioxidant enzymes expression following CCl_4_ administration [57, 58], others, including the current study, found increased levels after CCl_4_ challenge [59–61]. One probable cause of this increase was suggested to be the high free radical challenge that the enzymes encounter, leading to their overexpression [62]. Also, impairment of the bile flow result in the accumulation of toxic bile salts within the hepatocytes, with resultant injury caused by their detergent action. Also, bile salts can cause mitochondrial dysfunction by interfering with electron transport and hence H_2_O_2_ and superoxide formation. This causes SOD and CAT overexpression [63]. Another study pointed out that SOD expression increases in response to high nitrate levels [64].

Although liver injury caused firstly from the metabolism of CCl_4_ to the trichloromethyl radical and the resulted oxidative stress chain, secondary damage occurs from inflammatory processes that are initiated by Kupffer cells activation [65]. Activated Kupffer cells release some pro-inflammatory mediators that activate other cells in the liver (endothelial cells, stellate cells and hepatocytes), inducing chemokines expression which attract and activate circulating inflammatory cells [66]. One of these pro-inflammatory mediators is TNF-α [67]. The obtained results showed a marked increase in its levels in the CCl_4_ group, whereas *P*.*niruri* suppressed its expression. TNF- α stimulates the release of other cytokines from Kuppfer cells, in addition to induction of phagocyte oxidative metabolism and nitric oxide production [68].

NO, in turn, can exacerbate oxidative stress by forming peroxynitrite [69]. NO has pleiotropic effects. It is well documented that NO plays an important role in liver injury [70, 71], where the hepatocytes produce large quantities of NO [72] due to tissue damage and inflammation caused by a variety of xenobiotics as CCl_4_. Accordingly, data presented by various studies [73, 74] suggests a potential role of NO as an important mediator of CCl_4_-induced hepatotoxicity. The present results are in accordance with the above mentioned reports. High total NO levels were observed in the CCl_4_ group, which slowly approached normal levels by AE pretreatment.

The heightened inflammatory response was also evident by increased expression of COX-2 and iNOS. Cyclooxygenases are predominantly involved in inflammatory responses as they are the rate-limiting enzymes in prostaglandin (PG) biosynthesis from arachidonic acid. COX-2 is the inducible isoform that is responsible for PGs increased production in response to proinflammatory stimuli [75]. Thus, COX-2 expression inhibition is thought to be the most important target for assessing anti-inflammatory activities as it is specific to the inflamed tissue [76]. *P*. *niruri* resulted in decreased COX-2 expression.

Several reports have evidenced that overproduction of iNOS occurs in rats liver with CCl_4_-induced acute liver injury [73, 77] acting as a mediator in its pathogenesis [78]. Increased expression levels in the CCl_4_ group were confirmed by immunohistochemical staining which revealed inhibition of iNOS by AE.

Although Lee *et al*. [79] have previously reviewed the hepatoprotective potential of the hexane fraction and isolated compounds from *P*. *niruri*, they recommended further cheminformatics, toxicological and mechanistic studies in order to aid the progress of the plant to clinical trial studies. In the present work, the bioactive metabolites were isolated from the aqueous extract and further highlighted the anti-inflammatory properties of *P*. *niruir*, as evidenced by inhibition of COX-2, TNF-α, NF-KB, IL-6, IL-8 and IL10 as a possible mechanism of hepatoprotection in addition to the known antioxidant activities. The observed hepatoprotective effects of *P*. *niruri* AE may be due to the polyphenolic compounds. Further studies are needed in order to explore possible synergistic effects of different compounds present in the plant as well as their molecular mechanism working against hepatic damage.

## Conclusion

In conclusion, the obtained experimental data strongly support the view that *P*. *niruri* can be used as a promising hepatoprotective agent. Its activity can be attributed to its potent antioxidant and anti-inflammatory actions of its phenolic constituents. Reaching these positive findings seen in this article concerning the promising hepatoprotective activities of the aqueous extract and its constituents, it is recommended that preclinical studies on the herbal tea of the plant should be initiated.

## Supporting information

S1 Checklist NC3Rs ARRIVE guidelines checklist 2014. (DOCX)

S1 Table ^1^H NMR data of the isolated phytochemicals. (DOCX)

S2 Table ^13^CNMR data of the isolated phytochemicals. (DOCX)

S1 Minimal Dataset (XLSX)
