# Supplementary material for: In-depth hepatoprotective mechanistic study of Phyllanthus niruri: In vitro and in vivo studies and its chemical characterization
Source: PLoS One. 2020 Jan 15;15(1):e0226185. doi: 10.1371/journal.pone.0226185 (PMC6961881; doi:10.1371/journal.pone.0226185)

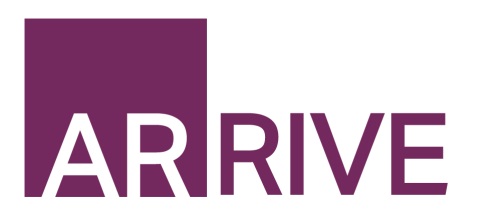


The ARRIVE Guidelines Checklist

Animal Research: Reporting In Vivo Experiments

Carol Kilkenny^1^, William J Browne^2^, Innes C Cuthill^3^, Michael Emerson^4^ and Douglas G Altman^5^

*^1^The National Centre for the Replacement, Refinement and Reduction of Animals in Research, London, UK, ^2^School of Veterinary Science, University of Bristol, Bristol, UK, ^3^School of Biological Sciences, University of Bristol, Bristol, UK, ^4^National Heart and Lung Institute, Imperial College London, UK, ^5^Centre for Statistics in Medicine, University of Oxford, Oxford, UK.*

|  | | ITEM | RECOMMENDATION | Section/ Paragraph |
| --- | --- | --- | --- | --- |
| 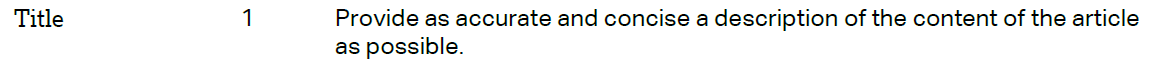 | | | Section 1 |  |
| 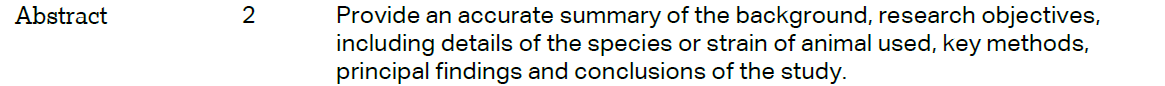 | | | Section 2 |  |
| INTRODUCTION | | |  |  |
| 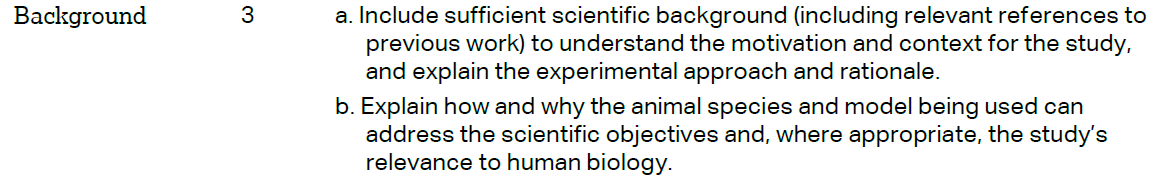 | | | Section 3 Pargraph 1,2 |  |
| 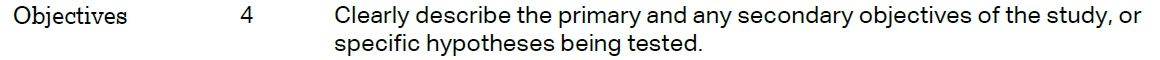 | | | Section 3 Paragraph 3 |  |
| METHODS | | |  |  |
| 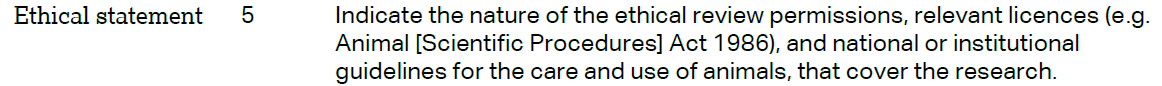 | | | Section 4 Paragraph 15 |  |
| 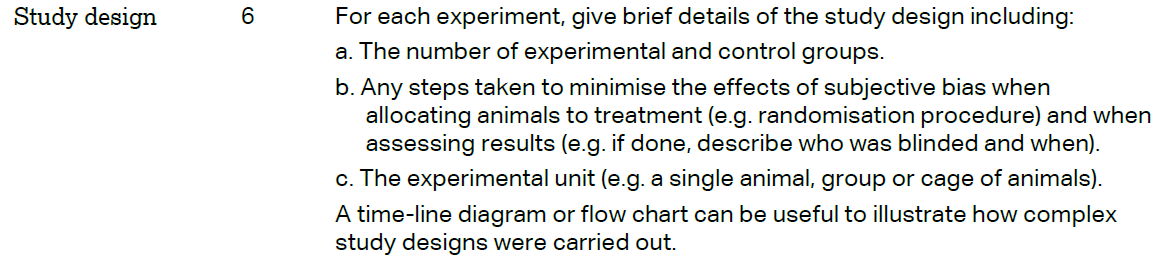 | | | Section 4 Paragraph 17 |  |
| 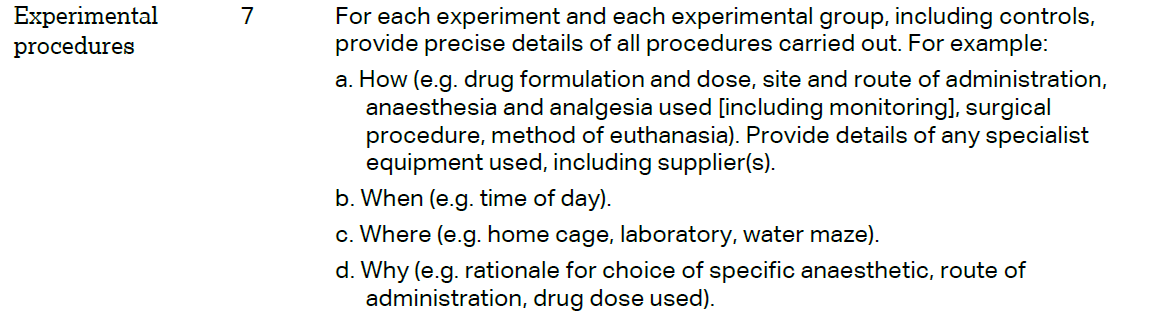 | | | Section 4 Paragraph 17 |  |
| 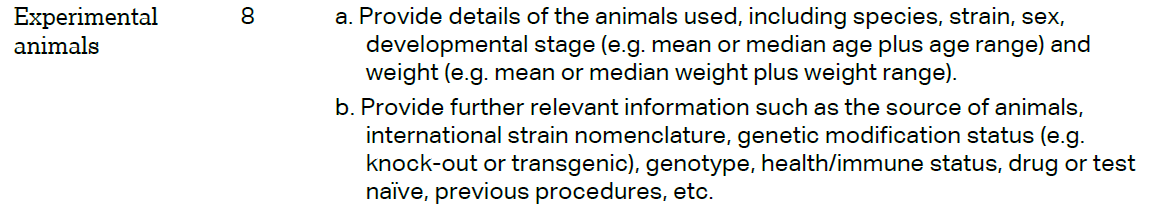 | | | Section 4 Paragraph 15 |  |

The ARRIVE guidelines. Originally published in *PLoS Biology*, June 2010^1^

| 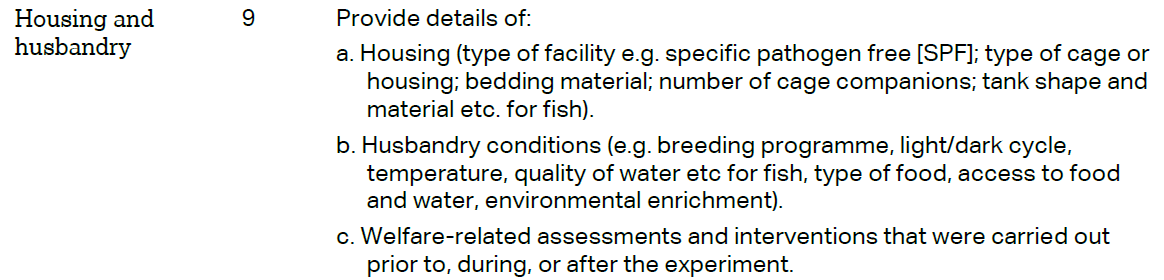 | Section 4 Paragraph 15 | |
| --- | --- | --- |
| 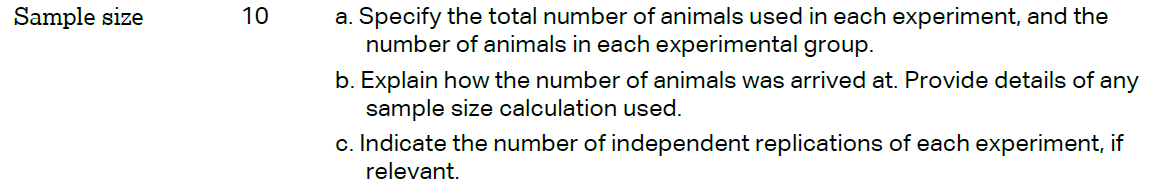 | Section 4 Paragraph 17 | |
| 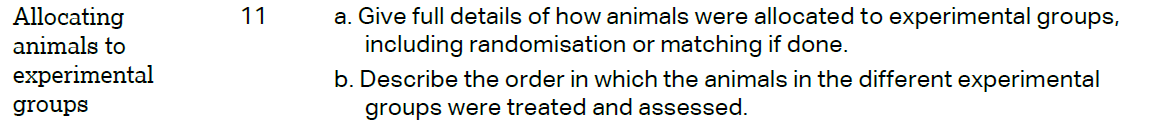 | Section 4 Paragraph 17 | |
| 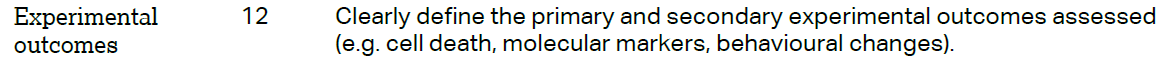 | Section 4 Paragraph 18-25 | |
| 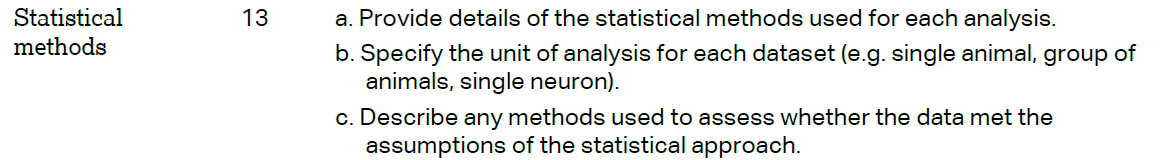 | Section 4 Paragraph 26 | |
| RESULTS |  | |
| 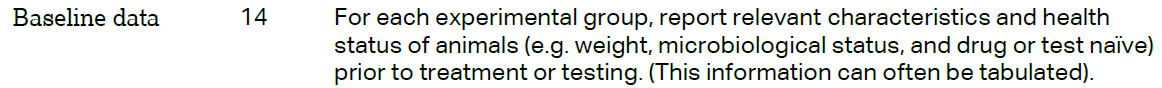 | Section 5 Paragraph 6 | |
| 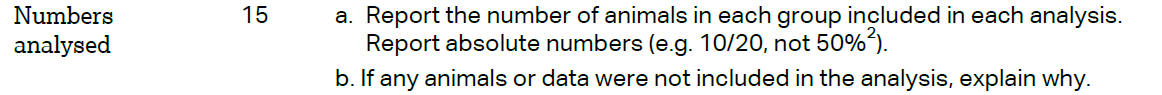 | Section 5 Paragraph 7-11 | |
| 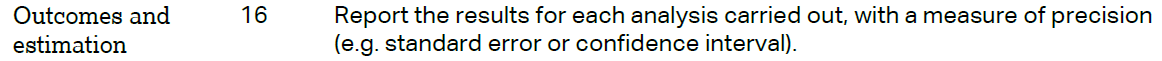 | Section 5 Paragraph 7-11 | |
| 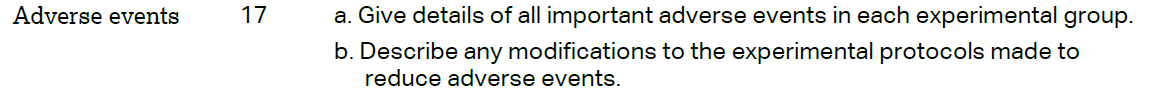 | Section 5 Paragraph 7-11 | |
| DISCUSSION |  | |
| 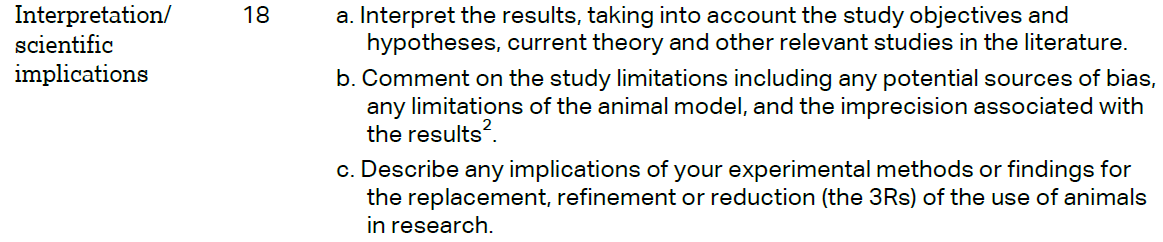 | Section 5 | |
| 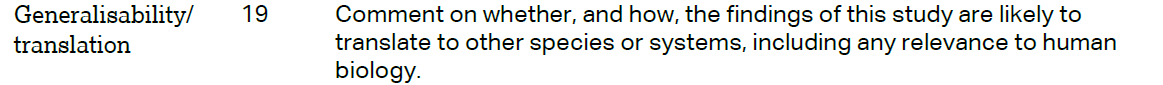 | Section 6 | |
| 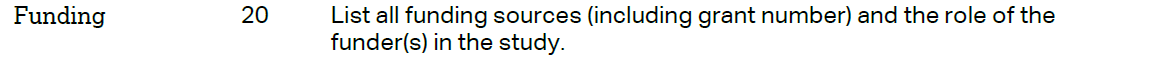 | | Section 11 |


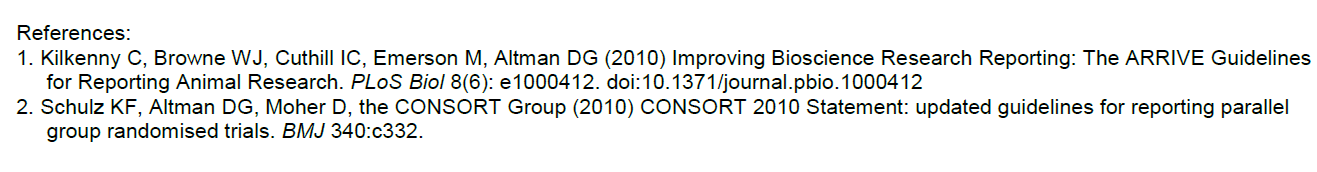

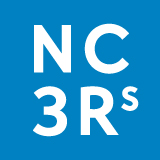

Supplement: S1 Checklist — (DOCX) [file pone.0226185.s001.docx]
