# Supplementary material for: In-depth hepatoprotective mechanistic study of Phyllanthus niruri: In vitro and in vivo studies and its chemical characterization
Source: PLoS One. 2020 Jan 15;15(1):e0226185. doi: 10.1371/journal.pone.0226185 (PMC6961881; doi:10.1371/journal.pone.0226185)
Supplement: S1 Table — (DOCX) [file pone.0226185.s002.docx]

**S1 Table: ^1^H-NMR data of compounds C1- C7 (^1^H 400MHz; *δ* in ppm, *J* in Hz)**

| **Position** | **C1 & C2** | **C3** | **C4** | **C5** | **C6** | **C7** |
| --- | --- | --- | --- | --- | --- | --- |
| **1** | 6.22 (1H, d, *J* = 7.5 for C1 & *J*= 2.2 for C2) | - | - | - | - | - |
| **2** | 3.89 (1H, d, *J* = 7.5 C1 & *J*= 2.2, C2) | - | - | - | 7.43 (1H, s) | - |
| **3** | 4.61 (1H, brs) | - | - | - | - | - |
| **4** | 4.28 (1H, br s) | - | - | - | - |  |
| **5** | 4.37 (1H, t, *J* = 8.1) | - | - | - | - | - |
| **6** | 4.24 (1H, dd, *J* = 10.8, 7.7, H-6a)  3.97 (1H, dd, *J* = 10.8, 10.2, H-6b) | - | 6.09 (1 H, d, *J*= 1.8) | 6.21 (1H, d, *J*= 2) | 7.43 (1H, s) | - |
| **7** | - | 7.3 (1H, s) | - | - | - | 7.29 (1H, s) |
| **8** | - | 2.51 (2H, m) | 6.29 (1H, d, *J*=1.8) | 6.41 (1H, d, *J*=2) | - | 4.42 (1H, dd; *J* = 7.7, 1.8) |
| **9** | - | 3.24 (2H, m) | - | - | - | 2.98 (2H, dd, *J* = 18.7, 7.7) |
| **2`** | 7.03 (1H, s) | - | 7.64 (1H, d, *J*=2) | 7.74 (1H, d, *J*=8.4) | - | - |
| **3`** | - | - | - | 6.9 (1H, d, *J*=8.4) | - | - |
| **5`** | - | - | 6.78 (1 H, d, *J*=8.4) | 6.9 (1H, d, *J*=8.4) | - | - |
| **6`** | 7.03 (1H, s) | - | 7.52 (1H, dd, *J*=2, 8.4) | 7.74 (1H, d, *J*=8.4) | - | - |
| **1``** | - | - | - | 5.28 (1H, brs) | - | - |
| **2``- 5``** |  | - | - | 3.07 – 3.66 (4H, m) | - | - |
| **6``** | 6.51 (1H, s) |  |  |  |  |  |
| **6```** | 6.58 (1H, s) | - | - | - | - | - |
| **CH_3_** | - | - | - | 0.78 (3H, d, *J* = 6) | - | - |
