# Supplementary material for: In-depth hepatoprotective mechanistic study of Phyllanthus niruri: In vitro and in vivo studies and its chemical characterization
Source: PLoS One. 2020 Jan 15;15(1):e0226185. doi: 10.1371/journal.pone.0226185 (PMC6961881; doi:10.1371/journal.pone.0226185)
Supplement: S2 Table — (DOCX) [file pone.0226185.s003.docx]

**S2 Table: ^13^C-NMR data of compounds C1- C7 (^13^C 125 MHz; *δ* in ppm)**

| **Position** | **C1 & C2** | **C3** | **C4** | **C5** | **C6** | **C7** |
| --- | --- | --- | --- | --- | --- | --- |
| **1** | 92.7 | 161.2 | - | - | 121.9 | 161.5 |
| **2** | 72.1 | 144.7 | 148.2 | 161.9 | 110.3 | 149.1 |
| **3** | 77.9 | 113.7 | 137.2 | 136.2 | 146.3 | 116.1 |
| **3a** |  | 141.9 |  |  |  | 140.1 |
| **4** | 62.6 | 145.5 | 177.5 | 179.5 | 139.5 | 146.7 |
| **5** | 76.8 | 140.6 | 162.6 | 163.2 | 146.3 | 140.7 |
| **6** | 64.4 | 149.8 | 99.4 | 99.8 | 110.3 | 143.4 |
| **7** | - | 108.5 | 165.7 | 165.9 | - | 108.4 |
| **7a** |  | 116.0 | - | - | - | 115.4 |
| **8** | - | 24.3 | 94.6 | 94.8 | - | 42.8 |
| **9** | - | 33.5 | 158.4 | 159.4 | - | 37.8 |
| **10** | - | 195.9 | 104.7 | 106.0 | - | 195.4 |
| **1՝** | 119.2 | - | 124.3 | 122.9 | - | - |
| **2՝** | 109.5 | - | 116.1 | 131.9 | - | - |
| **3՝** | 146.0 | - | 146.3 | 116.6 | - | - |
| **4՝** | 139.5 | - | 150.3 | 159.5 | - | - |
| **5՝** | 146.0 | - | 116.1 | 116.6 | - | - |
| **6՝** | 109.5 | - | 121.8 | 131.9 | - | - |
| **1``** | 116.3 | - | - | 103.6 | - | - |
| **2``** | 124.4 | - | - | 72.2 | - | - |
| **3``** | 145.2 | - | - | 72.1 | - | - |
| **4``** | 136.0 | - | - | 73.3 | - | - |
| **5``** | 144.8 | - | - | 72.0 | - | - |
| **6``** | 107.4 | - | - | - | - | - |
| **1```** | 116.0 | - | - | - | - | - |
| **2```** | 123.6 | - | - | - | - | - |
| **3```** | 145.3 | - | - | - | - | - |
| **4```** | 135.9 | - | - | - | - | - |
| **5```** | 144.4 | - | - | - | - | - |
| **6```** | 106.5 | - | - | - | - | - |
| **CH3** | - | - | - | 17.8 | - | - |
| **C=O** | 167.5  167.2 165.3 | - | - | - | 170.3 | 173.9 |
